# Supplementary material for: Predictors of self-management practices among diabetic patients attending hospitals in western Oromia, Ethiopia
Source: PLoS One. 2020 May 1;15(5):e0232524. doi: 10.1371/journal.pone.0232524 (PMC7194359; doi:10.1371/journal.pone.0232524)
Supplement: S1 File — (PDF) [file pone.0232524.s001.pdf]

Annex I: Information sheet

Dear Respondent, you are invited to participate in a research study to be conducted by instructors of Wollega University, College of Medical and Health Sciences. Please read the following statement and ask any unclear questions before you agree to participate.

1. **Objective of the Study:** To assess predictors of self-management practice among diabetic patients attending western Oromia hospitals, Ethiopia, 2017

The information you provide will help us to better understand what patients with diabetes practice to manage their own blood glucose level and diabetes related conditions. We would greatly appreciate your help in responding to this question. The result of the study would hopefully serve as an important input to intervention and policy programs aim at improving chronic illness care via launching the system of enhancing self-management support.

2. **Participation procedure and guideline:**
   a. The information you provide will be kept completely anonymous. Thus, your name will not be on any of the forms.
   b. FGD/In-depth interview will not take more than 1 hour.
   c. You may participate on FGD and in-depth interview questions
3. **Participation benefits and risks**
   a. Your participation in this study does not involve risks that are greater than those you experience in your daily life. Any information you give will be maintained confidentially.
   b. You may also experience some benefits from participating on this project which might be positive feelings from helping an important research study.
   c. No incentive or honorarium compensation will be given for participating in this study
4. Right to refuse or withdraw: Your participation is volunteer and there is no penalty for refusing to participate in the study. Thus, you are free to stop at any point or to choose not to answer any particular question or all the questions.
5. Right as a participant: You have a right to have questions about this research project. Please, direct any question to **Dereje Chala and Tariku Tesfaye.**
   a. Agree to participate? **Yes:** ☐ **No:** ☐

***We thank you in advance for taking your time to respond to our questions!**

Annex II: Consent form

I, the undersigned have been informed that the purpose of this particular research is to assess predictors of self management practice among diabetic patients attending western Oromia hospitals. I have also been informed that I will respond to the questions by answering what I know concerning the issue and the information I give will be used only for the purpose of this study. My identity and the information I provided will be kept confidentially. I have also gotten information that I can refuse to participate in the study or not to respond to questions I am not interested in. Furthermore, I have been informed that I can stop responding to the questions at any time in the process.

Based on the above information, I agree to participate in the research voluntarily with the hope of contributing (on behalf of one) to the effort of knowing status of DM self management practice and treatment outcome at_________________hospital.

Signature: _______________________ Date: ____________________________

Address of Investigators:

1. Dereje Chala (Principal Investigator)
   Cell phone: +251-913-34-26-27
   Email: derejechala@yahoo.com
2. Tariku Tesfaye (Co-Investigator)
   Cell phone: +251-920-23-37-98
   Email: tarii2007@gmail.com

Annex III: Questionnaire

Part 1: Socio- demographic characteristics of the respondents

No.	Questions& filters	Coding categories
Q1	Place of residence	Urban---------------1<br>Rural----------------2
Q2	Sex	Male---------------1<br>Female------------2
Q3	Age in years	_______
Q4	Religion	Protestant......1<br>Orthodox........2<br>Muslim ....3<br>Catholic--------4<br>Others...........5
Q5	Ethnicity	Oromo..........1<br>Amhara......... 2<br>Gurage---------3<br>Others...........4
Q6	Educational status	Can't read and write........1<br>1-4 grade.........................2<br>5-8 grade.........................3<br>9-12 grade------------------4<br>>12/College----------------5
Q7	Marital status	Never married/single---------1<br>Married--------------------------2<br>Divorced-------------------------3<br>Widowed-------------------------4<br>Others-----------------------------5

Q8	Occupation	House wife.........................1<br>Government employee.......2<br>Merchant...........................3<br>Student ....4<br>Local drink seller................5<br>House servant ....6<br>Daily laborer ....7<br>Other (specify).....................8
Q9	Average monthly income of the household in birr	<500...................1<br>500-999..............2<br>1000-1999.............3<br>2000 and above.....4<br>Don't know...........5
Q10	Compared to your neighbors, where do you classify your family's economical status?	Very poor...............1<br>Poor.......................2<br>Middle/average.....3<br>Rich.......................4<br>Very rich...............5
Q11	How many family members do you have now?	__________________
Q12	How are they related to you?	Husband/Wife------------1<br>Grandparents-------------2<br>Son/Daughter-------------3<br>Sister/Brother-------------4<br>Relative--------------------5<br>Adopted---------------------6<br>Home servant--------------7
Q13	Do you need your families' support in related to your diabetes disease?	Yes-------------------1<br>No--------------------2

Q14	Have your families ever supported you in related to your diabetes disease?	Yes-------------------1<br>No--------------------2
Q15	Do you have any chronic illnesses (Hypertension, HIV,Cancer and others)	Yes-------------------1<br>No--------------------2
Q16	Are there signs of DM Complication? (nephropathy, neuropathy, retinopathy, coma, heart disease)	Yes-------------------1<br>No--------------------2
Q17	Which type of Diabetes treatment are you receiving?	1. Insulin<br>2. Oral hypoglycemic agent<br>3. Both

Part II: Information related to Self-management

Q18	When were you diagnosed as Diabetic?	month______ year __________
Q19	Which type of diabetes do you have now?	Type 1-----------------1<br>Type 2-----------------2<br>Pre-diabetes----------3<br>Gestational-----------4<br>Don't know----------5
Q20	Have you had any previous instruction on how to take care of your diabetes?	Yes--------------------1<br>No---------------------2
Q21	Do you know about self-management in diabetes?	Yes-------------------1<br>No--------------------2
Q22	If Yes to Q21, Do you practice self-care?	Yes-------------------1<br>No--------------------2
	**Blood Glucose Test**	
Q23	If you practice self management, Do you check your blood glucose?	Yes-------------------1<br>No--------------------2

Q24	If yes to Q23, how often?	Once a day----------1<br>2 or more /day------2<br>1 or more/week-----3<br>Occasionally--------4
Q25	When do you check?	Before breakfast--------1<br>2 hours after meals-----2<br>Before bedtime---------3
Q26	What is your blood sugar range?	____________ to__________
Q27	In the last month, how often have you had a blood sugar less than 70?	Never-----------------------------1<br>Once------------------------------2<br>One or more__ times/week----3
Q28	Can you tell when your blood sugar is too high?	Yes-------------------1<br>No--------------------2
Q29	What do you do when your sugar is high?	______________________________
	**Diet/Meal Plan**	
Q30	Do you have a meal plan for diabetes?	Yes-------------------1<br>No--------------------2
Q31	If Yes to Q30, How often do you use this meal plan?	Never----------------1<br>Seldom--------------2<br>Sometimes----------3<br>Usually--------------4<br>Always--------------5
Q32	Do you read and use food labels as a dietary guide?	Yes------------------1<br>No-------------------2
Q33	Do you have any dietary restrictions?	Yes------------------1<br>No-------------------2
Q34	If yes to Q33, for which foods?	Salt------------------1<br>Fat-------------------2<br>Fluid----------------3<br>Others--------------4

Q35	Give a sample of your meals for a typical day:	1. Time:________Breakfast:_______<br>2.Time: ________ Lunch: _________<br>3.Time: _______ Dinner: _________<br>4.Time:________Snack: __________
	**Physical Exercise**	
Q36	Do you do physical exercise regularly?	Yes------------------1<br>No-------------------2
Q37	What type of exercise?	1. Participation in at least 30 minutes of exercise daily<br>2. Participation in a specific exercise session
	**Foot care**	
Q38	Do you practice Foot care?	Yes------------------1<br>No-------------------2
Q39	If Yes to Q38, what type of foot care?	1. Checking your feet<br>2. Inspecting the inside of your shoes

Thank you!!
