# Supplementary material for: Predictors of self-management practices among diabetic patients attending hospitals in western Oromia, Ethiopia
Source: PLoS One. 2020 May 1;15(5):e0232524. doi: 10.1371/journal.pone.0232524 (PMC7194359; doi:10.1371/journal.pone.0232524)
Supplement: S2 File — (PDF) [file pone.0232524.s002.pdf]

Afan Oromo version Questionnaire

I: Waraqaa Odeeffannoo

Seensa: Kabajamoo hirmaataa, qorannoo Yuunivarsiitii Wallaggaatti, barsiisota Kolleejjii Saayinsii Fayyaa fi Meedikaalaatiin geggeeffamu irratti akka hirmaattaniif haffeeramtaniittu. Itti hirmaachuuf murteessuu keessan dura, mee odeeffannoowwan armaan gadii dubbisaatii waan ifa isiniif hin taanes gaafadhaa.

1. **Kaayyoo Qorannichaa:** Hospitaalota dhiha Oromiyaa jiran keessatti namootni dhibee sukkaaraa qaban akka of hin yaalle (yaalii dhuunfaa akka hin goone) wantoota dhiibbaa geessisan qo’achuuf.

   Odeeffannoo isin nuuf laattan namootni dhibee sukkaaraa qaban sukkaara dhiiga isaanii keessa jiru to’achuufi tarkaanfiiwwan biro fudhachuuf maal akka godhan/shaakalan akka sirriitti hubannuuf baay’ee nu gargaara. Gaaffii keenyaaf deebii kennuuf tumsa isin nuuf gootaniif dursinee isin galateeffanna. Firiin qo’annoo kanaas sagantaawwanii fi poolisiiwwan dhibee gurguddoo umrii dheeraa namaa wajjin jiraatan kana fooyyessuuf keessattuu yaalii dhuunfaa jajjabeessaniif akka wabiitti ykn galiitti akka gargaaru itti amanama..

2. **Adeemsaa fi qajeelfama hirmaannaa:**
   a. Odeeffannoo isin laattan guutummaatti icitiidhaan qabama. Kunis, maqaan keessan unka kamiyyuu irratti hin guutamu/hin barreeeffamu.
   b. Mariiwwan gareedhaan ykn dhuunfaan geggeeffaman(FGD/IDI) turtii sa’a 1:00 olii hin fudhatan.
   c. Tarii mariiwwan gareedhaan ykn dhuunfaan geggeeffaman irratti hirmaachuu dandeessu.

3. **Bu’aa fi soda/miidhaa hirmaannaa**
   a. Qorannoo kana irratti hirmaachuun keessan miidhaa/sodaa jireenya keessan guyyaa guyyaa keessatti isin mudachuu danda’uu olitti waan isinitti fidu hin qabu. Odeeffannoo isin kennitan kamiyyuu icitiidhaan qabama.

b. Qorannoo kana irratti hirmaachuu keessaniif bu'aawwan tokko tokko argachuu dandeessu. Fknf Qorannoo barbaachisaa akkasii keessatti gumaacha keessan bahuun miira gaarii/namatti tolu isiniif uumuu danda'a.
c. Qorannoo kana irratti waan hirmaattaniif jedhamee kanfaltiin gosa kamiiyyuu kan hin jirre ta'uu kabajaan isin beeksifna.

4. **Mirga himaachuu dhiisuu/Addaan kutuu:** Hirmaannaan keessan fidhiidhaani. Jechuunis, qorannicha irratti hirmaachuu diduu keessaniif adabbiin hin jiru. Kanaafuu, yeroo barbaaddaniifi iddoo barbaaddanitti dhaabuuf/adda kutuuf ykn gaaffiiwwan gaafataman keessaa kan isin deebisuu hin barbaanne yoo jiraate deebisuu dhiisuuf mirga qabdu.
5. **Mirga akka hirmaataatti:** Qorannoo kan ilaalchisee gaaffii qabaachuun akkasumas deebii argachuun mirga keeti. Kana ilaalchisee Qorattoota kan ta'an B/saa **Darajjee Caalaa fi Taarikuu Tasfaayee** kallattiin gaafachuu dandeessu.
   a. Hirmaachuuf murteessitanii?? **Eeyyee:** ☐ **Lakki:** ☐

***Gaaffiiwwan keenyaaf deebii kennuuf yeroo keessan waan nuu laattaniif dursinee isin galateeffanna!**

II: Unka walii galtee

An, kanan armaan gaditti mallatteesse kaayyoon qorannoo kanaa hospitaalota dhiha Oromiyaa jiran keessatti namootni dhibee sukkaaraa qaban akka of hin yaalle (yaalii dhuunfaa akka hin goone) wantoota dhiibbaa geessisan qorachuuf ta'uusaa hubadheera.

Gaaffiiwwan dhiyaatan irratti waa'ee waanan gaafatamee waanan beeku akkan deebisuu fi odeeffannoo ani laadhus kaayyoo qorannoo kanaatiif qofa akka itti fayyadamamu hubadheera. Eenyummaankoo fi odeeffannoo ani kenne icitaan qabama. Akkasumas, qorannoo kana irratti hirmaachuu dhiisuuf ykn gaaffiin natti hin tolle yoo jiraate deebisuu dhiisuuf akkan mirga qabu natti himameera. Dabalataanis, adeemsa gaaffii fi deebii qorannichaa keessatti yeroo kamittuu addaan kutuu akkan danda'u naaf galeera.

Odeeffannoo armaan olii kana irratti hundaa'uudhaan, hospitaala________________tti yaaliin dhuunfaa namootni dhibee sukkaaraa qaban godhanii fi bu'aansaa maal akka fakkaatu keessatti gumaacha dhuunfaakoo akkan ba'achaa jiru abdachaa, qorannoo kana irratti fedhiikoon hirmaachuuf murteessuu koo nan mirkaneessa.

Mallattoo: ________________________ Guyaa: ______________________________

Teessoo Qorattootaa:

1. B/saa Darajjee Caalaa (Principal Investigator)

   Bilbila: +251-913-34-26-27

   Email: derejechala@yahoo.com

2. B/saa Taarikuu Tasfaayee (Co-Investigator)

   Bilbila: +251-920-23-37-98

   Email: tarii2007@gmail.com

III: Afan Oromo Questionnaire

Kutaa 1ffaa: Haalota hawaasaa fi dimogiraafii hirmaattotaa

Lakk.	Gaaffiiwwan	Deebii fi Koodiiwwansaanii
G1	Iddoo jireenyaa	Magaalaa---------------1<br>Baadiyyaa---------------2
G2	Saala	Dhiira---------------1<br>Dhalaa--------------2
G 3	Umrii (Waggaadhaan)	______
G 4	Amantii	Ortodoksii............1<br>pirootestaantii......2<br>Muusiliima...........3<br>Kaatolikii..............4<br>Kan biroo.............5
G 5	Saba	Oromoo............1<br>Amaara........... 2<br>Guraage---------3<br>Kan biroo.........4
G 6	Sadarkaa barnootaa	Dubbisuu fi barreessuu kan hin dandeenye.…….........1<br>Kutaa 1-4..................2<br>Kutaa 5-8..................3

		Kutaa 9-12............................4<br>>12/Kolleejjii........................5
G7	Haala Gaa'elaa/Bultii	Kan hin fuune/heerumne-----1<br>Kan fuudhe/heerumte---------2<br>Kan hike/hiikte------------------3<br>Kan irraa/jalaa du'e-----------4<br>Kan biroo------------------------5
G8	Hojii	Haadha manaa……...........1<br>Hojjetaa mootumma..........2<br>Daldaltuu...……………....3<br>Barattuuu……. ....4<br>Mashataa kan gurgurtu......5<br>Hojjettuu mana keessaa.....6<br>Dafqaaan bulaa………. ....7<br>Kan biroo …………..…...8
G9	Galii Giddu-galeessa maatii qarshiidhaan	<500.......................1<br>500-999..................2<br>1000-1999..............3<br>2000 fi isaa ol.........4<br>Hin beeku..............5
G10	Ollaa kee wajjin yeroo of madaalsistu kan armaan gadii keessaa kam keessatti of ramaddaa?	Baay'ee hiyyeessa...........1<br>Hiyyeessa........................2<br>Giddu-galeessa................3<br>Sooressa..........................4<br>Baay'ee sooresssa..........5
G11	Amma baay'inni/miseensi maatiikee meeqa?	________________
G12	Walitti dhufeenyi maatiikee fi kee karaa maaliiti/akkamiini?	Haadha/Abbaa warraa------1<br>Akkoo/Akaakayyuu-----------2<br>Ilma/Intala---------------------3<br>Obboleessa/ttii---------------4

		Fira----------------------------5<br>Guddifachaa------------------6<br>Hojjettuu mana keessaa---7
G13	Dhibee sukkaaraa keen walqabatee gargaarsa maatiikee ni feetaa/barbaaddaa?	Eeyyee--------------------1<br>Lakki----------------------2
Q14	Dhibee kanaan walqabatee maatiikee irraa deeggarsa/gargaarsa argattee beektaa?	Eeyyee--------------------1<br>Lakki----------------------2
Q15	Dhibeewwan biro qabduu? (Hypertension, HIV,Cancer and others)	Eeyyee--------------------1<br>Lakki----------------------2
Q16	Mallattoowwan sababa dhibee sukkaaraan dhufan kunneen jiruu? (nephropathy, neuropathy, retinopathy, coma, heart disease)	Eeyyee--------------------1<br>Lakki----------------------2
Q17	Wal'aansa/Yaala gosa kam fudhachaa jirtaa?	1. Insulin<br>2. Oral hypoglycemic agent<br>3. Lameenuu

Kutaa 2ffaa: Odeeffannoowwan wal'aansa dhuunfaan wal qabatan

G18	Dhibee sukkaaraa akka qabdu yoom barte?	Ji'a:______ Waggaa: __________
G19	Gosa dhibee sukkaaraa isa kami amma kan ati dhukkubsattu?	Gosa 1ffaa-------------------------1<br>Gosa 1ffaa-------------------------2<br>Dhibee sukkaara duraa---------3<br>Kan yeroo ulfaa nama qabu---4<br>Hin beeku------------------------5
G20	Dhibee sukkaaraa kee kanaaf akkamitti akka ofeeggachuu qabdu ajajni/gorsi siif kenname jiraa?	Eeyyee--------------------1<br>Lakki----------------------2
G21	Dhibee sukkaaraa keessatti waa'ee ofiin of wal'aanuu/yaaluu ni beektaa?	Eeyyee--------------------1<br>Lakki----------------------2
G22	Yoo deebiinkee **G21** eeyyee ta'e, ofiikeen of wal'aantee/yaaltee beektaa?	Eeyyee--------------------1<br>Lakki----------------------2
	**Sukkaara dhiiga keessaa ilaaluu**	

G23	Yoo of wal'aantee beekta ta'e, Hanga sukkaaraa dhiiga kee keessa jiru safartee beektaa?	Eeyyee--------------------1<br>Lakki----------------------2
G24	Deebiinkee G23[ffaa] 'Eeyyee' yoo ta'e, yoom yoom safarta?	Guyyaatti tokko----------1<br>Guyyaatti 2 ykn isaa ol------2<br>Torbanitti 1 ykn isaa ol-----3<br>Darbee darbee----------------4
G25	Yeroo akkam akkamii safarta/ilaalta?	Ciree dura------------------------------1<br>Nyaata nyaadhee sa'a 2 booda-----2<br>Yeroon rafuuf ka'u-------------------3
G26	Hangi sukkaaraakee meeqaa hanga meeqaa ture?	_____________ hanga ___________
G27	Ji'a darbe keessatti sukkaarri dhiiga kee keessaa yeroo meeqa 70 gadi ta'e?	Tasayyuu/gonkumaa---------------1<br>Yeroo tokko-------------------------2<br>Torbanitti yeroo 1 ykn isaa ol----3
G28	Yeroo sukkaarri dhiiga kee keessaa baay'ee oka'aa itti ta'e himuu ni dandeessaa?	Eeyyee--------------------1<br>Lakki----------------------2
G29	Sukkaarrikee yoo olka'aa ta'e maal goota?	________________________________
	**Waa'ee nyaataa/soorataa**	
G30	Dhibeekee kanaaf wantoota nyaattuuf karoora baastee qabdaa?	Eeyyee--------------------1<br>Lakki----------------------2
G31	Yoo qabda ta'e, karoora nyaataa kana yoom yoom fayyadamta?<br>How often do you use this meal plan?	Tasayyuu--------------------1<br>Darbee darbee--------------2<br>Yeroo tokko tokko--------3<br>Yeroo baay'ee--------------4<br>Yeroo hundaa--------------5
G32	Odeeffannoowwan soorata irratti barreffaman akka qajeelfama nyaataatti dubbistee itti gargaaramtaa?	Eeyyee--------------------1<br>Lakki----------------------2
G33	Nyaata keessaa kan ati irraa of qusattu/eeggattu ni jiraa?	Eeyyee--------------------1<br>Lakki----------------------2
G34	Deebiinkee G33[ffaa] 'Eeyyee' yoo ta'e, nyaata warra kamirraa of qusatta?	Ashaboo-------------------1<br>Cooma---------------------2

		Dhangala'aa---------------3<br>Kan biro--------------------4
G35	Mee naamunaa nyaata guyyaa tokko keessatti nyaattuu tarreessi.	1. Ciree:________ Sa'a:_________<br>2.Laaqana:_______ Sa'a: _______<br>3.Irbaata:_______ Sa'a: _________<br>4.Tursiistuu:_______ Sa'a: ________
	**Sosochii qaamaa**	
G36	Sosochii qaamaa walitti fufiinsa qabu ni gootaa?	Eeyyee--------------------1<br>Lakki----------------------2
G37	Sosochii qaamaa akkamii/gosa kam?	1. Sochii qaamaa yoo xiqqaate daqiiqaa 30 fudhatu.<br>2. Sochii qaamaa kutaa-kutaadhaan hojjetamu.
	**Sakatta'iinsa Miillaaf ta'u**	
G38	Miilla keef ofeegganoo/wal'aansa ni gootaa?	Eeyyee--------------------1<br>Lakki----------------------2
G39	Yoo deebiinkee G38, Eeyyee ta'e, ofeegganoo/wal'aansa akkamii goota?	1. Miilla ilaaluu/sakatta'uu<br>2. Keessa kophee sakatta'uu

Galatoomaa!!
