# Supplementary material for: Predictors of self-management practices among diabetic patients attending hospitals in western Oromia, Ethiopia
Source: PLoS One. 2020 May 1;15(5):e0232524. doi: 10.1371/journal.pone.0232524 (PMC7194359; doi:10.1371/journal.pone.0232524)
Supplement: Attachment — Submitted filename: Manuscript review feed back dm.docx [file pone.0232524.s005.docx]

Manuscript review feed back

PLOSE ONE

Manuscript number or ID: PONE-D-19-26947

Title: Predictors of self-management practice among diabetic patients attending western Oromia hospitals, Ethiopia

Reviewer’s report

Response on research problem

The study indentified some predictors of self care practice and magnitude of self care practice among DM patients in the stated study area which is important research area during this time when the problem related to chronic disease is getting worse in developing country than communicable disease. Despite its strength the manuscript has plenty of points and ideas need to be clarified further by authors. The result part need to be specific to objective and the way of report writing could be improved further in order to publish this paper on the other hand authors should incorporate operational definitions for measuring of important variables

In the title page section:

The author had stated the title as” *Predictors of self-management practice among diabetic patients attending western Oromia hospitals*, Ethiopia” the term western Oromia hospitals seems as if there were hospital called western Oromia hospitals and use same comment for this term throughout the manuscript

In abstract section:

Line 16 the sentence “Self-monitoring needs to be individualized and assist people with diabetes.” Has no connection with the idea of preceding sentence

Line 24 says SPSS version 20. But it is not advisable to use any abbreviation or acronyms in abstract unless described when used first

Line 26 there is another acronyms ‘DM’ which could have been described in this session again

Line 29 and 30 “The logistic regression analysis, merchants were seen to practice self-care 30 management about six times higher when compared to clients with other occupations” this sentence is meaningless or it seems as if logistic regression is being mention as one variable

Line 30 “(1.177-30.027 at 95% CI). “Too wide CI which may make the result of this study in doubt

Introduction section

Line 44 vs 46 the way the author cite the reference number is not uniform for example line 44 for ref no 1 author use’ ()’ but for others like ref no 8 the author use’ []’ . On the other hand it could have been better if the reference nos were written as (a,b,c) other than (a-c)

Line 48-54; there is paradox of idea in the paragraph “***number of diabetics in Ethiopia to be about 800,000 50 cases by the year 2000, and the number is expected to increase to 1.8 million by 2030” VS “It 51 was indicated later that there were over 2 million cases of diabetes in Ethiopia in 2014***” how could it be? The number was estimated to be increased to 1.8 million in 2030 but before 2030 the number was indicated to be 2million. This statement needs the author’s elaboration.

In addition to this; generally the introduction part of this study a little beat looks like literature review ; it lack coherent flow of idea and seems that author didn’t analyze the idea from different sources and report on his/her own word rather copied directly paragraph by paragraph from abstract of the studies

In introduction the authors could have indicated the gap or why the study was conducted but I could not find it here

Methods and materials section

In the study setting part; the authors did mention the important elements to be mentioned specific to this study: like number of target population (both tye 1 and 2 DM pts), number of health institutions giving service for DM patients (both governmental and nongovernmental)

Line 114-123; study participant. This part includes a number of sub topics under single topic. But it could have been better if each topic were written explicitly

Population (source and study popn); sample size cal, sampilig procedure etc )

The authors did not discuss the detail of sampling procedure to convince others to what extent that the data is reliable.

Line 126; the authors reported that the tool was adopted from different sources but fail to acknowledge the sources. In this part the author could have discussed the tool (part/sections of tool, no of items in each session)

The author did not mention any operational definition which is clue for the reviewer or reader to understand the finding of this study

The type of logistic regression analysis used; clear description of the type of models fitted (Bivariate and Multivariable) with the purpose; the cut of point (minimum p-value level)to declare statistical association between variables must be clearly indicated; the way of presenting the findings (out puts) of the bivariate and multivariable logistic regression models should be clearly stated; how variables were recruited for the final multivariable binary logistic regression model and how goodness of fit for the final model was tested should be clearly stated. Then one can make a simple judgment about the scientific validity and reputability of the analysis used by the authors of the manuscript

Line 151;it says ‘confidentiality will be ‘ better if changed to appropriate tense

Results

Line 158; “***The mean age of all respondents was 41.33 + 18.93 (SD) years***” SD could have been written as	‘+’

The author could have statistical ground in using mean, median etc which seems using mean for this study was not appropriate

Line 162,163, it says “The majority of the study participants 204 (51.3%) 163 ***came from urban and 194 (48.7%) from rural areas when they asked for their home town***” the term came from is not appropriate so I suggest the authors to use appropriate terminologies. Not only this but also for other sentences throughout the manuscript

Line 164; never married better if only ‘single ‘ as marital status is used

Line 169-173; says “When a patient's family’s ***economy compared 170 with their neighbor***, about 215 (54%) has a middle economy-level whereas about 122 171 (30.7%) are poor” how scientific is this finding? Unless known wealth quintile/ income cutoff point ; it is difficult report scientific finding like this

Line 176,177; it says ; “About 262 (65.8%) ever supported by their family and 36 177 (34.2) ***didn’t ever support” it seems as the DM patient need to give support for other***

***Line 179-181; “***Among patients who have the 180 disease, the majority of them 138 (93.8%) have ***hypertension*** and 9 (6.1%) ***have diseases 181 like cancer, edema, HIV/AIDS, dyspepsia, and bronchial asthma” need to clarified by authors because it is not clear how the investigator identified these health problem unless he has used observation checklist or hospital record which was not mentioned in data collection and tool part. The same is true for other finding from line 182-186***

***Abbreviations and acronyms were not described even in their first appearance like HIV/AIDS on line 181 and DKA on line 184***

***Line 202, 204, ‘says good approach and average’ but nothing was discussed about this measurement in operational definition. Let this alone even the author did not Incorporated operational definition for other important variable like ‘ self care practice’***

***The author could have to report finding about self care before predictors of self care practice but not***

***Line 222; it says ‘***knew about self –care management of which 129 (32.4%) are practicing it’ this also need further clarification from author how he/she measured knowledge or is all about ever heard …..

Line 248-252; the author drew conclusion here which could have been better if words for conclusion like high, low … were omitted and only the finding of cross tabulation is reported

Discussion section; discussion part need further revision and I suggest the author to limit the discussion only to self care practice its predictors

On the other hand it is better if the justification for the gap between this study and others will be scientific

Make discussion different from result

***Ref no 4, 12, 16, 17, 29 had no page number where as ref no 12, has no year of publication***

***Ref no 14 has no accessed date and website accessed at; ref no 15 is not acceptable totally***

***Ref no 38 need title***
