# Supplementary material for: Predictors of self-management practices among diabetic patients attending hospitals in western Oromia, Ethiopia
Source: PLoS One. 2020 May 1;15(5):e0232524. doi: 10.1371/journal.pone.0232524 (PMC7194359; doi:10.1371/journal.pone.0232524)
Supplement: Attachment — Submitted filename: Responses to Reviewers comment.docx [file pone.0232524.s006.docx]

**Point by point responses to Reviewers concerns.**

**Abstract:**

The methods are very short sighted to provide proper information. The result looks fragmented. The conclusion drawn not based on the finding?

**Response:** The methods are revised to the extent.

**Introduction:**

This section included literature review from different part of the world including Ethiopia. I found this necessary to make a strong argument in the discussion section. However, the write up looks very inconsistent and juggling from the beginning to the end. You should follow introduction writing guide line. Since, the topic is highly researched in various part of Ethiopia I recommend authors to incorporate as many literature from Ethiopia as possible.

**Response:** Thank you for your constrictive idea. It’s fully revised and corrected as per the suggested ideas.

**Methods:**

I have noticed similar study had been conducted one year prior to this study in Nekemet, Western Oromia region. What is your rationale to Nekemete Hospital in this study?

**Response:** Yes, previously there was a study conducted 6 years back. The justification for conducting this study was: 1^st^, after 6 years, there are so many changes in contexts, social, political and other demographic aspects. Secondly, this study covers a wider public health facility with completely different geographic areas with different setups. This enables this study to identify the problem under consideration with wider contexts with different population parameters.

How you operationally defined the study variables in this research? What is your dependent and independent variables?

**Response:** The methods are completely revised and the operational definition which was missed is now included in the manuscript as one **component of methods**

How you developed the questionnaire? How you validate your questionnaire? Where you pretested the questionnaire?

**Response:** This is addressed now and the detail can be checked in the revised manuscript with changes highlighted uploaded with this response.

**Result:**

The socio-demographic section seems to have included junk of information and redundancy. You must take account of socio-demographic variables than unrelated information. In general, the result section is full of in consist, in comprehensive and fragments of data. Most of the paragraph appears to have a copy and paste. I would recommend the authors to give appropriate time to evaluate the manuscript from the beginning to the end before submission to make sure the information built-in is clear and vivid information to understand.

Response: We noted that the result was written inconsistently and not organized well. We have rewrite the result.

**Discussion:**

As it has been said this topic is one of the most studied, hence the chance to get appropriate literature to make a strong argument on the finding is likely very high even in Ethiopia. However, the arguments are shallow, not based on evidence and weak. Why you cited literature which doesn’t have any similarity to the study setting, for instance America and others. This makes the discussion very shallow and inadequate.

Response: Discussion part is revised and discussed by comparing with available evidences.

**What is the limitation of this study?**

# “The causal relationship could not be established due to nature of the study (cross-sectional). Self-management practice was measured based on clients’ self-response which might have not represented the actual practice due to social desirability bias.”

# It’s already included in the manuscript immediately next to discussion.

**Conclusion:**

How do you measure whether self-care is good or bad? There is no single statement in the methods section that clearly defined self-care and how it was measured. Overall, the conclusion not inferred from the analyzed result.

**Response:** It’s now revised as per your constructive suggestion.

Data Availability statement

We declare that we can provide SPSS data available.

**Additional Editor Comments (if provided):**

**Reviewers' comments:**

Reviewer's Responses to Questions
**Comments to the Author**

3. Have the authors made all data underlying the findings in their manuscript fully available? **NO**
4. Is the manuscript presented in an intelligible fashion and written in standard English? **NO** 
**Reviewer #1:**

**Response:** Thank you very much for your constructive messages/comments! Introduction part is completely re-written from the beginning accordingly.

**Reviewer #2:**

**Response:** Abstract section is fully revised as per your suggestion. Thank you very much.
**Result**

Response: Thank you for your suggestion. Results were re-written.

**Discussion:**

**Response:** Thank you for your suggestion. Discussion was discussed by incorporating the literature.
**Reviewer #3:**

**Response:** The whole manuscript is revised for grammatical, editorial and other errors you suggested. Thank you very much!
