# Supplementary material for: Predictors of self-management practices among diabetic patients attending hospitals in western Oromia, Ethiopia
Source: PLoS One. 2020 May 1;15(5):e0232524. doi: 10.1371/journal.pone.0232524 (PMC7194359; doi:10.1371/journal.pone.0232524)
Supplement: Attachment — Submitted filename: Response to Reviewers last.docx [file pone.0232524.s007.docx]

**Response to reviewers**

**Question 1**: Did you evaluate knowledge based on individual question mean or the summation mean of the knowledge questions?

**Response**: Thank you for thorough evaluation and observation. We didn’t evaluate the diabetes knowledge score; however, we reported the general knowledge about diabetes management. Thus, we have removed from the operational definition part.

**Question 2.** Secondly, the argument used to discuss the finding seems to be so frail and not enough to provide vivid clarification. In general, this section needs to have literary supported evidence with clear references or citations for your argument.

**Response:** Thank you for your comment. We noted that our discussion is weak, thus we made modifications with implications of the results. We also cited the literature we have used.
