# Supplementary material for: Predictors of self-management practices among diabetic patients attending hospitals in western Oromia, Ethiopia
Source: PLoS One. 2020 May 1;15(5):e0232524. doi: 10.1371/journal.pone.0232524 (PMC7194359; doi:10.1371/journal.pone.0232524)
Supplement: Attachment — Submitted filename: reviewer comments on predictors of self managment among diabetic patient.docx [file pone.0232524.s004.docx]

**Abstract:**

- Under method section number of study participants needs to be included
- Under result number of those who have either good or poor self care management needs to be indicated.
- The conclusion should be in line with the pertinent finding described under abstract
- Recommendation is not in line with conclusion.

**Introduction**

- Definition of self care management needs to be included.
- References needs to be in logical order.

**Methods**

The study setting is not well described.

Tools used to assess self care management was not clearly indicated.

Analysis method for self care management is not indicated.

Under ethical clearance future tense was used.

**Result**

- Standard deviation of mean age should be reported in plus or minus, not only plus
- Under table 1 you put asterisk on the other category of religion, but not define it below the table as footnote.
- Eye problems which is considered as one complication of DM is indicated by two different numbers.
- Rather than including other information better narration follows similar table.
- There is redundancy of narration about patients believe of diabetic treatment before and after **table 2**
- Under statements which is written above the title “**predictors of self management**” under result section, there is a statement which says **figure 2,** but there is no figure in the document.
- Better you include all variable you consider for logistic regression to table 3 with their respective confidence interval and odds ratio rather than duplicating it under table 4
- How you found at the end two predictor variable using logistic regression is not clearly explained.
- Revise your interpretation of logistic regression especially your comparison of occupation.
- How self care management is measured?????????? And classified as good and poor under the title “**level of self care practice”** are not clearly indicated.

**Discussion**

Your discussion needs revision. Implications given by the researcher is not satisfactory.

**Conclusion**

The conclusion is not in line with that of the title of the study and there are repeated copying and pasting of what is already described under result section.

**Reference**

Some of the references listed are not utilized for preparation of the manuscript

Finally competing interest and list of abbreviations/acronyms are not included to the manuscript
